# Proposed international concept for instrument testing

**DOI:** 10.1155/S1463924684000183

**Published:** 1984

**Authors:** R. Haeckel

**Affiliations:** Zentralkrankenhaus St.-Jürgen-Strasse Bremen Germany


Journal of Automatic Chemistry, Volume 6, Number 2 (April–June 1984), pages 88–90

### Proposed international concept for instrument testing

**R. Haeckel**
*Zentralkrankenhaus, St.-Jürgen-Strasse, Bremen, FR Germany*

The current world-wide economic recession has meant that the decision-making process for purchasing new instrumentation has gained more importance and priority for laboratory managers.

The process requires careful consideration of cost and, even more important, comparable data on the reliability and dependability of a particular instrument. At the present time it is often very difficult to obtain objective, comparable data. When a new item of equipment comes on the market a prospective purchaser often has little information about its performance, other than that from the manufacturer, or perhaps a colleague who might have done a trial for the manufacturer. Neither of these sources could be considered independent and it is unlikely that any testing will have followed a carefully designed and internationally accepted protocol. Therefore, many laboratories perform extensive evaluation studies before a purchase decision is made.

Standardization of evaluation studies would improve the comparability of the results reported. Several national and international organizations are involved in standardizing the procedure for instrument testing, especially for evaluations of analytical analysers. Some examples are listed in table 1. These protocols are all similar; therefore, they should be combined into one internationally acceptable recommendation. However, it appears that it is difficult to produce a single document to serve everyone's purposes. At least three evaluation stages have been identified for an instrument new to the market. Each stage has its own purpose, so a different protocol for each is required (these are presently elaborated by various organizations).

*Table 1. Evaluation protocols proposed by various organizations.*

| *Proposed evaluation standards* | *Organization* |
|---|---|
| Recommended scheme for the evaluation of instruments for automatic analysis in the clinical biochemistry laboratory | British Society for Clinical Chemistry [1] |
| Protocol for establishing performance claims for clinical chemical methods | NCCLS [2] |
| Recommendations for multi-centre evaluations of analytical instruments | German Society for Clinical Chemistry [3] |
| Protocol for the evaluation of analytical instruments | ECCLS [4] |
| Protocol for the evaluation of automatic blood-cell counters | ICSH (not yet published) |
| Provisional guidelines for a short-term assessment for inter-laboratory acceptance of clinical laboratory instrumentation | IIFCC (not yet published) |

The first stage (see table 2) is completely in the responsibility of the manufacturer. The experiments can either be performed in the factory or in external users' laboratories. It is common for manufacturers to ask one or several routine laboratories for an evaluation with various goals:

(1) To detect weak points, since routine conditions are hard to simulate in a company's laboratory.
(2) To solve possible problems in sample- and data-handling.
(3) To obtain an external publication which is considered a useful advertisement.
(4) To establish performance criteria.

The American National Committee for Clinical Laboratory Standards (NCCLS) has published several documents for this purpose.

Evaluations organized by a manufacturer are usually regarded as an essential part of a responsible quality-control programme for the production process. External evaluations are presumably less cost-effective than internal studies with comparable expenditure. No time scale can be suggested for stage 1 because modifications of the instrument are often required.

Lists of specifications, which should be provided by the manufacturer, have been worked out and published by the International Federation of Clinical Chemistry's (IFCC) Expert Panel on Instrumentation (EPI) for analysers and several other types of instrumentation (table 3). It is to be hoped that these guidelines will soon be adopted by manufacturers and customers.

*Table 2. Stage 1 evaluation.*

| | |
|---|---|
| *Primary goal*: | establishment of performance criteria |
| *Responsibility*: | manufacturer |
| *Production line*: | prototype(s) of instruments for the zero/first production line |
| *Location*: | no recommendation |
| *Time required*: | no recommendation |
| *Document prepared* by NCCLS | |

*Table 3. Recommendations by the IFCC's Expert Panel on Instrumentation for listing specifications by the manufacturer.*

| |
|---|
| Provisional guidelines for listing specifications |
| of spectrometers (see references [8, 9 and 10]) |
| of analysers [11] |
| of flame-emission spectrometers [12] |
| of atomic absorption spectrometers [13] |
| of nephrelometers (in preparation) |
| of blood-gas analysers (in preparation) |
| of ion-selective electrodes (in preparation) |

The principal goal of stage 2 is to verify the specifications which have been set up during stage 1 (table 4). During a 1978 workshop conference organized by the Commission of the European Communities under the title 'A plan for the use of national resources for evaluating instruments employed in clinical laboratory sciences', the idea of a co-ordinated multi-centre evaluation trial was proposed as a way of achieving comparability between the results from different laboratories. Such multi-centre evaluations should last for no more than three to six months.

*Table 4. Stage 2 evaluation.*

| | |
|---|---|
| *Primary goal*: | verification of performance criteria established during stage 1 |
| *Responsibility*: | some users in consultation with the manufacturer |
| *Production line*: | instruments from the first, or, even better, from later production lines |
| *Location*: | several users' routine laboratories |
| *Time required*: | three to six months |
| *Document prepared* by ECCLS/ICSH | |

*Table 5. Stage 3 evaluation.*

| | |
|---|---|
| *Primary goal*: | checking of performance criteria (established during stage 1 and verified in stage 2) |
| *Responsibility*: | all users |
| *Production line*: | from any production line |
| *Location*: | user's laboratory |
| *Time required*: | about two weeks |
| *Document prepared* by EPI of IFCC | |

This concept has been adopted by a working group of the German Society for Clinical Chemistry. The document published by this group has been revised by the *ad hoc* Committee for Instrument Testing [4] of the European Committee for Clinical Laboratory Standards (ECCLS); the concept is summarized in figure 1. Some specifications (safety and technical specifications for example) are verified simply by an auditing of the manufacturer, others by performing experiments in clinical laboratories.

Although special attention was paid to the statistical part of the document, several comments received show that it is difficult to find a proposal which suits all possible users. There seems to be general agreement that classical regression analysis should be abandoned in favour of either standardized principal component analysis [5] or another non-parametric procedure [6]. Further attention has been paid to the presentation and reduction of the numerous data obtained during a multi-centre evaluation.

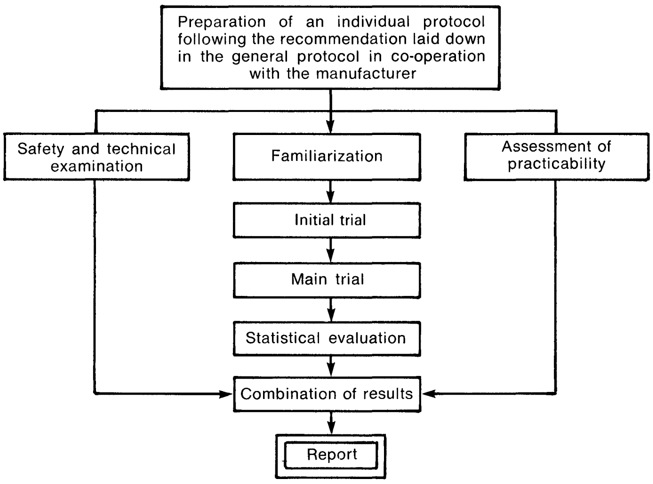

*Figure 1. Multi-centre concept for the evaluation of analysers according to a draft proposal of ECCLS.*

In stage 1, performance claims can be obtained by following the NCCLS protocols especially designed for this purpose. It may also be possible to use parts of the multi-centre protocol to determine precision, accuracy, carry-over, etc., in either the manufacturer's own and/or external laboratories. The laboratories establishing and verifying the claims should not be identical, some overlap, however, may be acceptable. Data obtained from a multi-centre evaluation may later be used by the manufacturer when performance characteristics are published.

The third and last stage (table 5) is intended for all instruments made on a production line. This evaluation should be performed by all laboratories before a new instrument is used for the analysis of samples from patients, to check whether the performance criteria established and verified in stages 1 and 2 still hold. Evaluation studies in stage 3 require only about two weeks.

The three-stage concept was first suggested by the European groups [4 and 7] but it is also proposed in a draft document recently distributed by the International Committee for Standardization in Haematology (ICSH). This document has many features in common with the ECCLS draft standard:

(*a*) Multi-centre trials.
(*b*) Definition of tolerance limits: prior to the evaluation the acceptable limits of precision and accuracy should be stated. These will depend on the manufacturer's claims and on clinical requirements.
(*c*) Familiarization period.
(*d*) Safety assessment.

Other proposals in the ICSH document which are not covered by the ECCLS standard are:

(i) Participation in national external quality-assessment schemes.
(ii) Details for calibration procedures on quality control materials and for sample collection.

Differences exist in terminology concerning imprecision and in statistical procedures.

The concept of three stages more or less reflects the present procedure adopted by larger companies introducing a newly developed analyser. Although this procedure is already followed in many cases, it is important that the concept is generally accepted before standardization proceeds further. Since voluntary trials are proposed, the concept is not considered as a pre-market approval procedure.

It is proposed that only a few laboratories should conduct standardized and co-ordinated multi-centre evaluations; their results would then be used by most laboratories for selecting new instrumentation. For a final decision only a short testing period of two weeks is required.

Experiences with the present situation have shown that single-centre evaluations are not always accepted, especially outside the country in which they were performed; and that instrument companies seldom use only one laboratory for their evaluation studies. This situation can be called 'multiple one-centre evaluations'. The disadvantages are that the time involved before reports from several sites are published is considered to be over long; the data produced on the various laboratories are often not comparable because differences exist in experimental design; the number of evaluations required by European users is usually considered to be too high by the manufacturers; and there is much wastage of personnel and scientific resources.

Therefore, the next step would be to co-ordinate several trials in one multi-centre evaluation. Major advantages of this concept are:

(1) Comparable data on the reliability of new instruments from several laboratories would be produced.

(2) Information about new instruments would be more easily accessible.
(3) The data would be more objective because the results would be more independent of the manufacturer. The degree of objectivity obtained by a multi-centre study cannot be reached by single laboratories.
(4) Unnecessary replication of numerous evaluations would be avoided.
(5) Expert and financial resources would be used optimally.
(6) Instrument improvement would be stimulated.

Advantages (1), (2) and (3) improve the basis for decision-making on purchases; (4) and (5) mean that less manpower and cost would be required from the manufacturer. Advantage (6) creates improved feedback of laboratory experience to the manufacturer. The data are much more meaningful if they are obtained in several laboratories under comparable conditions.

Assuming that the three-stage concept is accepted, the next step should be harmonization of the various protocols already elaborated concerning terminology and basic features to be tested.

This harmonization has already been achieved between the present version of the stage 2 document published by ECCLS and the stage 3 document elaborated by the IFCC's EPI. No attempts have been made to harmonize these documents with the stage 1 documents worked out by NCCLS. This has led to some misunderstandings. The NCCLS documents have been used for stage 2 purposes; similarly, the ECCLS stage 2 protocol could be used as a guideline for stage 1.

Experiences with the present ECCLS protocol have shown that several specific documents are required for each class of instrument, for instance for spectrometers, chemical analysers, blood-gas analysers and so on.

In conclusion, the three-stage concept for testing new instrumentation should generally be accepted and the performance of multi-centre evaluations should be endorsed on a voluntary basis.

### References

1. Broughton, P. M. G., Gowenlock, A. H., McCormack, J. J. and Neill, D. W. J., *Clinical Biochemistry*, **11** (1974), 207.
2. National Committee for Clinical Laboratory Standards Proposed Standard: PSEP-3 (1979). Protocol for establishing claims for clinical chemical methods (USA).
3. Haeckel, R., Kller, H. and Knedel, M., *Comm. Germ. Soc. Clin. Chem.*, **5** (1980), 184.
4. Haeckel, R., Busch, E. W., Christiansen, F. F., Gentilini, J. L. and Jennings, R. D., ECCLS document **2**, No. 4 (1982).
5. Feldmann, U., Schneider, B., Klinkers, H. and Haeckel, R., *Journal of Clinical Chemistry and Clinical Biochemistry*, **19** (1981), 121.
6. Passing, H. and Bablok, W., A new biometrical procedure for testing the equality of measurements from two different analytical methods. *Journal of Clinical Chemistry and Clinical Biochemistry*, **21** (1983), 709.
7. Haeckel, R., *Advances in Clinical Chemistry*, **1** (1982), 255.
8. Haeckel, R., Collombel, Ch., Geary, T. D., Mitchell, F. L., Nadeau, R. G. and Okuda, K., *Clinical Chimica Acta*, **103** (1980), 249.
9. Haeckel, R., Collombel, Ch., Geary, T. D., Mitchell, F. L., Nadeau, R. G. and Okuda, K., *Journal of Clinical Chemistry and Clinical Biochemistry*, **18** (1980), 445.
10. Haeckel, R., Collombel, Ch., Geary, T. D., Mitchell, F. L., Nadeau, R. G. and Okuda, K., *Clinical Chemistry*, **27** (1981), 187.
11. Okuda, K., *Journal of Clinical Chemistry and Clinical Biochemistry*, **18** (1980), 947.
12. Bechtler, G., Epstein, M. S., Geary, T. D., Havemann, W. and Attoe, P., *Journal of Clinical Chemistry and Clinical Biochemistry*, **20** (1982), 259.
13. Epstein, M. S., Geary, T. D., Gower, G., Tausch, W., Mills, K. J. and Polt, D., *Journal of Clinical Chemistry and Clinical Biochemistry*, **20** (1982), 263.

### AUTOMATIC CHEMICAL ANALYSIS

The 6th Summer School of Automatic Chemical Analysis was held at the University of Sussex, Falmer, Brighton, UK from 1 to 6 July 1984. Key topics were automation, computing, and laboratory management. The Summer School is intended to enable analysts from a wide range of industries to acquire the basic skills needed for laboratory automation; experienced users of automated systems should have their knowledge and appreciation of the latest developments in the field improved.

Each year the organizers of the School evaluate participants' comments and consider ways of improving the course. Consequently, the 6th School emphasized automated chemistry and contained less on computing. The proven format of an integrated series of lectures, tutorials and practical sessions was retained.

The emphasis on automated chemistry was reflected in both the lecture topics and the list of lecturers and tutors; new names included Professor Ray Dessy from Virginia State Polytechnic, USA, and Professor Jarda Ruzicka from the Technical University of Denmark.

*Information about the 1985 School from Beverly Humphrey, Summer School of Automatic Chemical Analysis, 176a North View Road, London N8 7NB, UK.*

### NEW JOURNAL

*Food Additives and Contaminants*

Taylor & Francis Ltd, publisher of *Journal of Automatic Chemistry*, launched a new quarterly called *Food Additives and Contaminants* at the beginning of the year.

The journal contains original contributions and review articles relating to the detection, determination, occurrence, persistence, safety evaluation and control of naturally occurring and man-made additives and contaminants in the food chain.

Subjects covered include:

- Naturally occurring toxins
- Residues of environmental contaminants, including pesticides
- Substances added during food and beverage processing
- Contaminants arising from packaging and storage
- Naturally occurring—or added—vitamins, hormones, antibiotic or drug residues
- The effects resulting from enzymic or radiation processes
- Surveillance and indentification of groups at risk
- Implications of national and international legislation on food quality
- Chemical form and other factors affecting bioavailability.

The editors are Drs R. Walker and M. E. Knowles. *Enquiries (1984 prices are £37·00, $74·00 and DM 167·00) to Taylor & Francis Ltd, Rankine Road, Basingstoke, Hampshire RG24 0PR, UK.*

